# Supplementary material for: Promoting Sports Practice in Persons with Hemophilia: A Survey of Clinicians’ Perspective
Source: Int J Environ Res Public Health. 2021 Nov 11;18(22):11841. doi: 10.3390/ijerph182211841 (PMC8625842; doi:10.3390/ijerph182211841)
Supplement: Supplementary file 1 [file ijerph-18-11841-s001.zip › ijerph-1399624-supplementary.pdf]

SCREENING

S1. Doctor, you practice as….

1. HEMATOLOGIST
2. PEDIATRIC HEMATOLOGIST
3. SPORTS PHYSICIAN
4. Other → *close the survey*

S2. Do you work in regione PUGLIA?

1. YES
2. No → *close the survey*

ONLY IF S1- HEMATOLOGIST/PEDIATRIC HEMATOLOGIST

S3. In which Hemophilia center do you work?

____

S4. Can you declare your age?

|__|__| years

S5. How long have you practiced as __(*S1*)?

|__|__| years

QUESTIONAIRE

TO ALL

D1. Thinking of an average year, how many patients do you care, for any disease?

Number of patients |__|__|__| __|

TO ALL

D2. Among these ___ (D1) patients, how many have any type of hemophilia?

Number of patients with hemophilia |__|__| __|

TO ALL

D3. Among___ (D2) patients with hemophilia, how many have each of the following types of hemophilia?

1. Number of patients with hemophilia type A |__|__|__|
2. Number of patients with hemophilia type B |__|__|__|

Total=D2

TO ALL

D4. The ___ (D2) patients with hemophilia, how are they divided into age classes?

1. % Patients with hemophilia younger than 6 years |__|__|__| %
2. % Patients with hemophilia 7-12 years old |__|__|__| %
3. % Patients with hemophilia 13-18 years old |__|__|__| %

4. % Patients with hemophilia older than18 years |__|__|__| %

Total 100%

TO ALL

D5. In average, how many times do you visit a patient with hemophilia in one year?

Mean number of visits/year/patient |__|__|

TO ALL

D6. During the visits of patients with hemophilia, which subjects do you address? Which are the subjects that you believe must be communicated/shared with the patient?

……

ONLY IF S1-HEMATOLOGIST/PEDIATRIC HEMATOLOGIST

D7. And especially, if the visit time is 100, how much time (%) is taken by each of the following subjects?

1. Prevention of bleedings |__|__|__| %
2. Counseling about lifestyle and everyday activities |__|__|__| %
3. Check of adherence to therapy |__|__|__| %
4. Pain management |__|__|__| %
5. Counseling about exercise and sports |__|__|__| %
6. More (Indicate______________________) |__|__|__| %

Total 100%

Considering only sports and patients with hemophilia

TO ALL

D8. Are you aware about existing guidelines/protocols specific for exercise and sports for patients with hemophilia?

1. Yes, they exist
2. No, they do not
3. I am not

IF YES D8

D9. Which guidelines do you mean? Which ones do you know? Can you briefly describe them?

____

TO ALL

D10. Do you have protocols for the physical activity and sports in patients with hemophilia, in your center?

1. Yes
2. No

IF YES D10

D11. What is recommended by these protocols? Can you briefly describe the protocols?

TO ALL

D12. You are going to read some statements about sports and hemophilia. Please declare how much you agree with them on a 1 to 10 scale, where 1= "not at all in agreement" e 10= "in complete agreement"

	**Score 1-10**
1) I always encourage my patients with hemophilia to practice regular physical activity/sports	
2) It is mandatory that patients with hemophilia practice regular physical activity to improve their psychological wellbeing and their emotional and social dimension	
3) It is mandatory that patients with hemophilia practice regular physical activity/sport for the wellbeing of muscles and bones.	
4) A regular exercise helps to improve stability and joint functionality, reduces the risk of acute bleedings and their complications.	
5) A regular exercise improves the overall quality of life of patients with hemophilia	
6) Patients with hemophilia in the developmental age should practice regular exercise/sport since the beginning of their disease, to improve stability, joint functionality, and bone density and to reduce the risk of bleedings.	
7) The practice of physical activity/sport is not indicated for all patients with hemophilia.	
8) It is not easy to encourage patients to practice sports, because parents are concerned and afraid.	
9) Only some sports are suitable for patients with hemophilia.	

TO ALL

D13. According to your experience, which are the main limits and barriers to the practice of physical activity or sport (either competitive or not) for patients with hemophilia?

……

TO ALL

D14. Among patients with hemophilia who do not practice exercise or sport, which are the main barriers?

1) The patient is afraid of possible injuries/bleedings
2) The patient thinks that control visits and monitoring are too demanding
3) The patient thinks that the process to obtain the fitness certificate is too long and demanding
4) The patient has severe hemophilia
5) The family/caregivers disagree, oppose
6) It is difficult to obtain the fitness certificate from the Sports Medicine Service
7) The patient wishes to practice sports which are not adequate to his/her condition
8) Lack of communication about real opportunities for the practice of sports
9) Other, describe_______________________________________

TO ALL

D15. Among the ___ (D2) patients with hemophilia you see, how many directly ask for counsel/support to practice regular exercise/sport?

% Patients with hemophilia who ask to practice sport |__|__|__| %

TO ALL

D16. And how many among the ___ (D2) patients with hemophilia you see, actually practice regular exercise/sport?

% Patients with hemophilia regularly practice sports |__|__|__| %

TO ALL

D17. And among these patients with hemophilia who practice regular exercise/sport, how many of them practice competitive sports and how many non-competitive sports?

% patients with hemophilia practice non-competitive sport |__|__|__| %

% patients with hemophilia practice competitive sport |__|__|__| %

Total 100

TO ALL

D18. And these patients with hemophilia who practice regular exercise/sport, how are they divided into age classes?

1. % Patients with hemophilia regularly practice sports, are younger than 6 years |__|__|__| %
2. % Patients with hemophilia regularly practice sports, are 7-13 years |__|__|__| %
3. % Patients with hemophilia regularly practice sports, are14-18 years |__|__|__| %
4. % Patients with hemophilia regularly practice sports, are older than18 years |__|__|__| %

Total 100

TO ALL

D19.Which are the 3 sports you usually recommend to a patient with hemophilia, and which are the 3 sports you usually advise against?

BETTER RECOMMENDED SPORTS	ADVISED AGAINST SPORTS
1)	1.
2)	2.
3)	3.

ONLY IF S1-SPORTS PHYSICIAN

D20. Which test/clinical examination do you usually prescribe a patient with hemophilia asking for the fitness certificate, either for competitive activity or non-competitive activity?

A. TO ASSESS ABILITY TO COMPETITIVE SPORTS	B. TO ASSESS ABILITY TO NON-COMPETITIVE SPORTS
1) Medical history	Medical history
2) Blood pressure measure	Blood pressure measure
3) Resting ECG	Resting ECG
4) Stress ECG	Stress ECG
5) Spirometry	Spirometry
6) Urine test	Urine test
7) Vision test	Vision test

8) Other, describe ______________________	Other, describe ______________________

ONLY IF S1-SPORTS PHYSICIAN

D21. When you must release a fitness certificate to one of your patients with hemophilia, you

A. IF COMPETITIVE SPORT	B. IF NON-COMPETITIVE SPORT
1) I ALWAYS refer him/her to other specialists for further evaluations	I ALWAYS refer him/her to other specialists for further evaluations
2) I SOMETIMES refer him/her to other specialists for further evaluations	I SOMETIMES refer him/her to other specialists for further evaluations
3) I NEVER refer him/her to other specialists for further evaluations	I NEVER refer him/her to other specialists for further evaluations

ONLY IF S1-SPORTS PHYSICIAN
IF CODE 1 OR 2 FOR A, or B -D21

D22. And usually, which are the other specialists you consult before you release a fitness certificate to the patient with hemophilia?

1. Orthopedic
2. Cardiologist
3. Hematologist of the center for hemophilia
4. Physiatrist
5. Physiotherapist
6. Other, describe______________

ONLY IF S1-SPORTS PHYSICIAN

D23. How long does it usually take to have all necessary tests and release the fitness certificate to a patient with hemophilia?

____________**days** *OR*
____________ **months**

ONLY IF S1-SPORTS PHYSICIAN

D24. How many times do you visit a patient with hemophilia to release a fitness certificate?

____________**number of visits**

ONLY IF S1-SPORTS PHYSICIAN

D25. During the visits of a patient with hemophilia for the fitness certificate, which subjects do you address? (more than one answer is possible)

1) I advise the most suitable sport
2) I advise strategies to prevent the bleeding risk
3) I explain which visits and controls he/she must attend periodically
4) Other, describe____

TO ALL

D26. Once the patient with hemophilia has started exercise/sport, how often do you control him/her?

Every ____________

A TUTTI

D27. On your experience with patients with hemophilia who want to practice exercise/sport, which are the main obstacles you face? Which are the areas and the steps that need improvement in the management of these patients?

___

TO ALL

D28. How much do you agree that each one of the following proposals about the patient with hemophilia practicing exercise/sports should be implemented? Show how much you agree with each one, by a1 to 10 score, where 1= "not at all in agreement" e 10= "in complete agreement"

1. Providing an insurance	
2. Require a signed informed consent from the patient/caregiver	
3. compliance to hemophilia prophylaxis	
4. infusion of the clotting factor before the physical activity	
5. Any sport must be practiced safely (make available a toll free number for the hemophilia center/coach-caregiver, who should be adequately trained for emergencies)	

TO ALL

D29. I am going to read some statement about sports and patients with hemophila. Show how much you agree with each one, by a1 to 10 score, where 1= "not at all in agreement" e 10= "in complete agreement"

	**Voto 1-10**
1) The visit to assess the fitness to the practice of either competitive or non-competitive sports must be made by the specialist in Sports Medicine, after reception of documentation of the joint condition and of compliance to the therapy prescribed by the hematologist of the hemophilia center	
2) A special service should be activated for people with hemophilia who want to start a regular physical activity, to receive requests of fitness assessment, and follow-up the clinical and administrative process needed to issue a certificate	
3) Non-competitive sports may, not needing a fitness certificate, may be advised by the hematologist of the hemophilia center, after consulting the sports physician	
4) National and Regional Health Services should provide a multidisciplinary team (hematologist + sports physician + muscle and bone specialist) to evaluate the requests of fitness assessment	
5) The hematologist of the hemophilia center should personalize the therapy on the basis of the request to start a regular physical activity, of the clinical conditions of the patient, and of requirements issued by the sports physician	
6) Telemedicine (real time video-consult with the hemophilia specialist during the competition or the training with the possibility to share a clinical record and test results) may be a facilitating tool for the certification of fitness and or the beginning of a correct physical activity	
